# Widespread Detection of Multiple Strains of Crimean-Congo Hemorrhagic Fever Virus in Ticks, Spain

**DOI:** 10.3201/eid2802.211308

**Published:** 2022-02

**Authors:** María Paz Sánchez-Seco, María José Sierra, Agustín Estrada-Peña, Félix Valcárcel, Ricardo Molina, Eva Ramírez de Arellano, Angeles Sonia Olmeda, Lucía García San Miguel, Maribel Jiménez, Luis J. Romero, Anabel Negredo, Group for CCHFv Research

**Affiliations:** CIBER Enfermedades Infecciosas (CIBERINFEC), Madrid, Spain (M.P. Sánchez-Seco, M.J. Sierra, R. Molina, A. Negredo);; Universidad de Zaragoza, Zaragoza, Spain (A. Estrada-Peña);; Instituto Nacional de Investigación y Tecnología Agraria y Alimentaria, Madrid (F. Valcárcel);; Instituto de Salud Carlos III, Madrid (E. Ramírez de Arellano, M. Jiménez);; Universidad Complutense de Madrid, Madrid (A.S. Olmeda);; Ministerio de Sanidad, Consumo y Bienestar, Madrid (L. García San Miguel);; Ministerio de Agricultura, Alimentación y Pesca, Madrid (L.J. Romero)

**Keywords:** Crimean-Congo hemorrhagic fever, hemorrhagic fever infections, Hyalomma lusitanicum, arboviruses, tick-borne viruses, surveillance, genetic variability, viruses, Spain, vector-borne infections, zoonoses

## Abstract

Human cases of Crimean-Congo hemorrhagic fever (CCHF) were first detected in Spain in 2016. National human and animal health authorities organized a large, multidisciplinary study focusing on ticks as sentinels to determine the nationwide distribution of ticks with CCHF virus. Ticks were collected from animals and vegetation, samples pooled (12,584 ticks; 4,556 pools), and molecular methods used to look for the virus. We detected the virus in 135 pools from most of the regions studied, indicating that it is widespread in Spain. We found sequences of CCHF virus genotypes I, III, and IV in the tick species collected, most commonly in *Hyalomma lusitanicum*, suggesting this tick has a prominent role in the virus’s natural cycle. The red deer (*Cervus elaphus*) was the host that most frequently yielded positive ticks. Our study highlights the need for larger studies in Spain to ascertain the complete risk to public health.

Crimean-Congo hemorrhagic fever (CCHF) is a tickborne zoonotic disease causing severe illness, considered by the World Health Organization to be 1 of the 7 highest-priority epidemic-prone diseases. CCHF is considered the most widespread tickborne viral hemorrhagic disease in the world and poses a great public health risk because of its epidemic potential, high case-fatality rates in humans, and a lack of effective mitigation measures, creating an urgent need for accelerated research (*1*). CCHF is caused by CCHF virus (CCHFV; family *Nairoviridae*, genus *Orthonairovirus*), a negative-sense single-stranded RNA virus. The virus is a spherical virion 80–120 nm diameter and has a lipid envelope. Its genome is divided into 3 segments; the small (S) segment encodes the viral nucleocapsid, the medium (M) segment the membrane glycoprotein precursor, and the large (L) segment the RNA-dependent RNA polymerase protein (*2*). The S segment has been widely used in phylogenetic studies, which have defined 6 of 7 CCHFV lineages, each with a different geographic range (*3*,*4*).

The virus is transmitted to humans mainly by the bite of infected *Hyalomma* spp. ticks, which act as reservoirs and vectors; sexual, transovarial, and transstadial transmissions have been demonstrated in the ticks (*5*). Immature ticks commonly feed on medium-sized mammals and on birds, but adults prefer domestic and wild ungulates, which do not develop clinical signs; reports of the tick on other vertebrates are anecdotal (*6*,*7*). The virus can also be transmitted by direct contact with infected fluids of animals and humans. Groups at risk include farmers and their families, slaughterhouse and healthcare workers, veterinarians, and persons who are otherwise prone to being bitten by ticks (*8*).

CCHFV has been widely reported across the whole of Africa, except for the Sahara Desert, and in Asia and Europe (*9*,*10*), where its range overlaps with that of its main tick vectors. Human CCHF cases in Europe had usually been reported in countries of the former Soviet Union and some Balkan countries before 2 human clinical cases were detected in Spain in 2016 (*11*), raising awareness of the virus’s circulation in western Europe. The index case-patient was bitten by a tick while walking in a field in Ávila province, belonging to the Castile and León (CyL) region (autonomous community) (Figure 1). The second case was a nosocomial infection in a healthcare worker. Since then, 8 additional cases have been described in Spain: 1 in 2013 (documented recently in a retrospective study), 2 in 2018 (1 found retrospectively), 3 in 2020, and 2 in 2021 (*12*–*16*). Epidemiologic tracking of these human cases revealed a wider distribution of the virus than initially expected. The patient in 1 of the 2018 cases was infected in Badajoz province in the Extremadura (EXT) region; all other patients were infected in CyL: 2 in Avila, 1 in León, and 5 in Salamanca provinces (Figure 1). Strikingly, the viral genotypes detected in human cases were highly variable. Genotype III (Africa 3 clade) was found in cases from 2016 and 2020 (*11*,*17*; A. Negredo, pers. comm., email, 2021 Sep 30). Cases detected in 2018 consisted of CCHFV genotype V (Europe 1 clade) (*14*); a reassortment of genotype IV (Africa 4 clade) in the S segment; and genotype III (Africa 3 clade) in the M and L segments (*13*).

**Figure 1:**
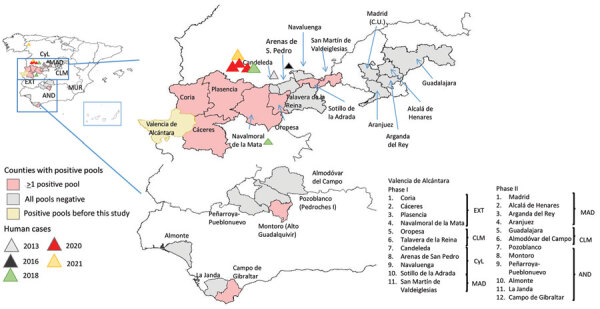
Local distribution of sampling areas in study of Crimean-Congo hemorrhagic fever virus in ticks, Spain. Pink indicates areas where CCHFV was detected in tick pools during this study; triangles indicate human cases. Inset shows locations of sampling areas in Spain. CyL, Castile and León; CLM, Castile-La Mancha; MAD, Madrid; EXT, Extremadura; AND, Andalusia; MUR, Murcia

In Spain, the virus was first detected in 2010 in *H. lusitanicum* ticks collected while they were feeding on red deer (*Cervus elaphus*) in Cáceres province, EXT region (*18*). Additional surveys detected the virus in the same area over several years, and sequencing revealed that several variants of genotype III (Africa 3 clade) were circulating there (*19*,*20*). Recently, RNA of genotypes IV (Africa 4 clade) and V (Europe 1 clade) have also been detected in ticks collected from red deer and wild boar (*Sus scrofa*) in several regions of southwestern Spain (*21*).

Our study reports the results of an extensive CCHFV surveillance study in Spain, involving more than 12,000 ticks collected while they were questing or feeding. The aim was to provide an up-to-date overview of the current distribution and genetic variability of the CCHFV strains found so far in Spain, with a special focus on future risk assessment.

## Materials and Methods

For this study, we considered ticks to be sentinels of CCHFV distribution in Spain. Questing or feeding ticks were collected in 2 different phases from wild and domestic animals across a wide geographic area of Spain during October 2016–2018 (Figure 1). In the first phase, which lasted 6 months (October 2016–March 2017), ticks were collected from livestock flocks in 11 counties in 4 regions, CyL, Madrid (MAD), Castile-La Mancha (CLM), and EXT, where *Hyalomma* ticks were already known to be present (Figure 1). Earlier, smaller surveys had established the presence of CCHFV in both ticks and humans (*11*,*18*,*20*). Adult ticks were collected mainly from wild large game ungulate species such as red deer, wild boar, fallow deer (*Dama dama*), and mouflon (*Ovis orientalis musimon*) and barbary sheep (*Ammotragus lervia*), as well as from grazing livestock cattle (*Bos primigenius taurus*) and goats (*Capra aegagrus hircus*) that had not recently received an ixodicidal treatment and had been grazing on farms for ≥30 days. A maximum of 25 ticks per animal were collected; all ticks were removed from animals on which <10 ticks were found. In small herds of wild ungulates, all of the animals were sampled, but only 10%–15% of the members of larger herds were sampled.

Ticks from the same animal were kept alive in separate, labeled sterile vials and transported at ambient temperature with controlled humidity until they could be frozen at –80°C when possible, then sent to the Spanish National Centre of Microbiology (NCM) for tick identification and viral molecular analysis. Ticks were identified using taxonomic keys (*22*,*23*), and pools were produced with ≤4 fed ticks (depending on size and food status) with specimens collected from the same animal and of the same tick species. Ticks collected in CLM were identified at local laboratories and then sent to NCM. Ticks of the same species collected from different animals were combined in 1 tube. 

In the second phase, during May–September 2017 and March–July 2018, ticks were collected from vegetation by standard flagging in 15 counties in 4 regions where the circulation of the virus had not previously been described: MAD, CLM, Murcia (MUR), and Andalusia (AND) (Figure 1). After ticks were collected and transported to the laboratory, they were morphologically identified (*22*,*23*), then samples were frozen and sent to NCM for molecular processing. Pools were produced with 3 ticks obtained from the field.

### RNA Extraction

We washed ticks 2× with water and 1× with 70% ethanol, then pooled and crushed them using a plastic homogenizer in a mixture of 560 μL AVL buffer (QIAGEN, https://www.qiagen.com) and 140 μL water. We extracted RNA as described elsewhere (*20*).

### Molecular Identification

We performed real-time reverse transcription PCR (RT-PCR) as described elsewhere, but with slight modifications (*14*,*24*), to amplify the 1–122 region of S segments as the screening method. To confirm results, we used a nested RT-PCR that amplifies the 123–764 region in the first amplification and the 450–674 region in the second amplification in the S segment (*11*). We considered a pool positive when both PCRs were positive or when one of the PCR tests gave positive results from 2 different extracts. We sequenced amplicons and performed phylogenetic analysis in a 175 bp fragment of the S gene, as described elsewhere (*20*). We deposited sequences ≥200 nt long obtained with primers CriCon1+ and CriCon1– (*11*) in the European Molecular Biology Laboratory and GenBank databases (accession nos. OK 082060–OK 082067). For Caceres 2140 SPN 2016, we obtained only 1 sequence (CGTCAATGCAAATACAGCAGCCCTAAGCAACAAAGTCCTCTCTGAGTACAAGGTTCCTGGTGAGATTGTGATGTCTGTCAAAGAGATGCTCTCAGACATGATCAGAAGGAGGAATCTGATCCTTAACAGAGGGGGTGATGAGAACCCAAGGGGCCCAGTAGGCAAGGAGCATATA), which was <200 nt long, so we did not deposit it in the database.

## Results

A total of 12,584 ticks were collected and pooled, 3,959 pools (10,793 ticks) from animals (Tables 1, 2, 3) and 597 pools (1,791 ticks) from vegetation (Table 4). Adult *H. lusitanicum* were predominant among ticks collected while feeding, but we also recorded *H. excavatum*, *H. marginatum*, *H. rufipes*, *Dermacentor marginatus*, *Ixodes ricinus*, *Haemaphysalis punctata*, *Rhipicephalus annulatus*, *R. bursa*, *R. pusillus*, and *R. sanguineus* sensu lato ticks (Table 1). We identified all but 2 questing ticks as *H. lusitanicum*.

**Table 1:** Ticks collected from ungulates and percentages of the different species identified in each region in study of Crimean-Congo hemorrhagic fever virus in ticks, Spain

Tick species	Madrid, %, n = 230	Castile and León, %, n = 829	Extremadura, %, n = 7,917	Castile-La Mancha, %, n = 1,817
*Dermacentor marginatus*	6.3	12.8	7.1	23.2
*Haemaphysalis punctata*	0	1.5	0	0.7
*Hyalomma excavatum*	9.4	0	0	0
*H. lusitanicum*	81.3	8.3	70.1	55.8
*H. marginatum*	0	2.3	0.6	1.3
*H. rufipes*	0	0	0.1	0
*Ixodes spp*	0	0	0	5.7
*I. ricinus*	0	24.1	8.0	7.3
*Rhipicephalus annulatus*	3.1	44.4	14.0	0
*R. bursa*	0	6.8	0.1	4.4
*R. pusillus*	0	0	0	0.1
*R. sanguineus*	0	0	0	0.5

**Table 2:** Ungulates sampled for tick collection and testing to determine the presence of Crimean-Congo hemorrhagic fever virus in ticks, Spain

Region (province)	Wild ungulates, no. (%)							Domestic ungulates, no. (%)		
	Red deer	Wild boar	Fallow deer	Mouflon	Barbary sheep	Total		Cattle	Goats	Total
Extremadura (Cáceres)	671 (3.2)	161 (1.2)	12 (0)	12 (0)	2 (0)	858 (2.8)		166 (0)	1 (0)	167 (0)
Madrid (Madrid)	16 (6.2)	7 (14.3)	2 (0)	3 (33.3)	0 (0)	28 (10.7)		0 (0)	0 (0)	0 (0)
Castile and León (Toledo)	44 (0)	8 (0)	5 (20.0)	0 (0)	0 (0)	57 (1.7)		69 (0)	7(0)	76 (0)
Total	731 (3.1)	176 (1.7)	19 (5.3)	15 (6.7)	2 (0)	943 (2.9)		235 (0)	8 (0)	243 (0)

*Percentages indicate animals in which Crimean-Congo hemorrhagic fever virus was detected in feeding ticks.

**Table 3:** Positive pools of ticks and genotypes of CCHFV, according to small segment sequences, detected in ticks collected while they were feeding on ungulates, Spain*

Region (province)	No. pools (no. ticks)	No. (%) positive pools	Tick species found with CCHFV (no. pools)	Animals found with CCHFV-infected ticks (no.)	Genotypes†
Madrid (Madrid)	90 (230)	7 (7.7)	*Hyaloma lusitanicum* (6), *Dermacentor marginatus* (1)‡	Mouflon (1), wild boar (1), red deer (1)	IV
Castile and León (Avila)	338 (829)	1 (0.3)	*Rhipicephalus annulatus* (1)	Fallow deer (1)	IV
Castile-La Mancha (Toledo)	642 (1,817)	76 (11.8)	*H. lusitanicum* (76)	Red deer§	III
Extremadura (Cáceres)	2,889 (7,917)	44 (1.5)	*H. lusitanicum* (42), *Ixodes ricinus* (2)¶	Red deer (22), wild boar (2)	I, III, IV
Total	3,959 (10,793)	128 (3.2)	NA	Red deer (>23), wild boar (3), fallow deer (1), mouflon (1)	I, III, IV

*CCHFV, Crimean-Congo hemorrhagic fever virus; NA, not applicable. †Genotypes: I, West Africa (Africa 1); II, Central Africa (Africa 2); III, South and West Africa (Africa 3); IV, Middle East/Asia, divided into groups Asia 1 and Asia 2; V, Europe/Turkey (Europe 1); VI, Greece (Europe 2). ‡Collected from wild boar. §In Castile-La Mancha, ticks of the same species but collected from different animals were mixed in a single tube, so it was not possible to determine the number of animals with positive ticks. ¶Collected from red deer.

**Table 4:** Genotypes of Crimean-Congo hemorrhagic fever virus, according to small segment sequences, detected in host-seeking adult *Hyalomma lusitanicum* ticks collected from vegetation, Spain*

Region	Province	No. pools (no. ticks)	No. (%) positive pools	Genotype
Andalusia	Huelva	113 (339)	0	NA
	Cádiz	66 (198)	5 (7.6)	III
	Córdoba	103 (309)	2 (1.9)	IV
Castile- La Mancha	Guadalajara	99 (297)	0	NA
	Ciudad Real	37 (111)	0	NA
Madrid	Madrid	146 (438)	0	NA
Murcia	Murcia	33 (99)	0	NA

*NA, not applicable.

### Feeding Ticks

We collected ticks from 1,186 ungulates in CyL, MAD, and EXT: 943 wild and 243 domestic animals (Table 2). Red deer (n = 731) was the most common host species, followed by wild boar (n = 176), fallow deer (n = 19), and mouflon (n = 15) and barbary sheep (n = 2). Among livestock, we surveyed cattle (n = 235) more often than goats (n = 8). No ticks feeding on livestock were positive for CCHFV. Excluding animals from the CLM region, for which data were not available, we found positive ticks on 2.9% (28/943) of all surveyed wild animals.

**Figure 2:**
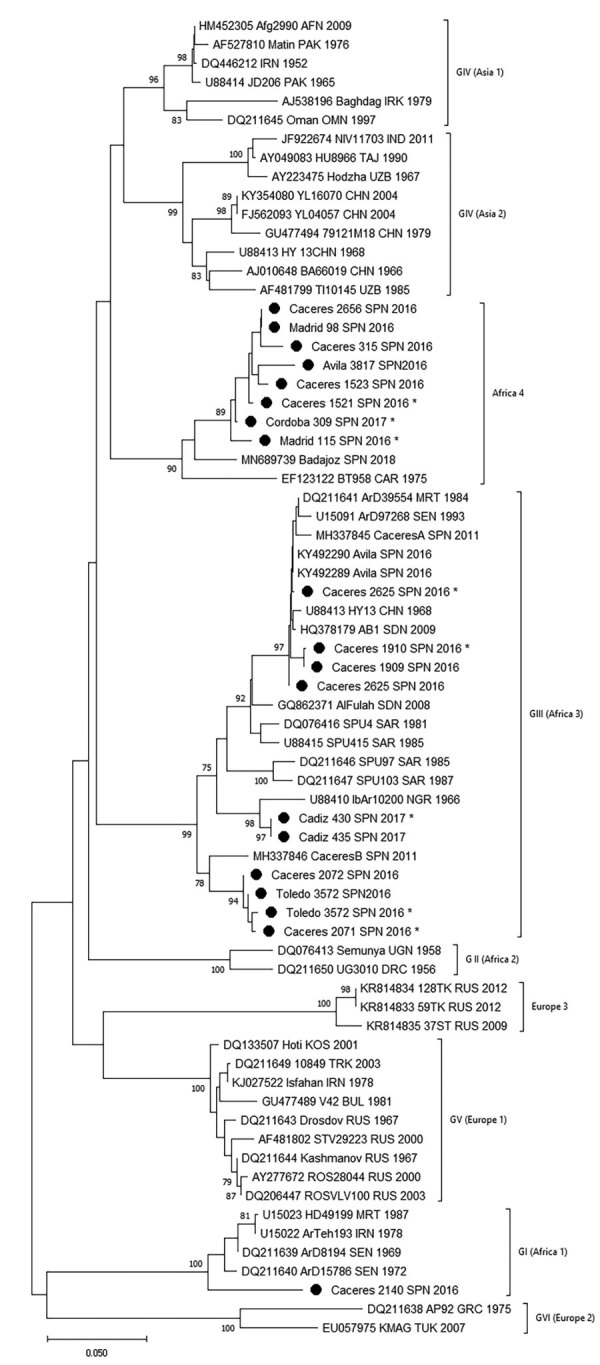
Phylogenetic tree obtained for strains of Crimean-Congo hemorrhagic fever virus detected in Spain (black dots) and other sequences downloaded from GenBank. We built the phylogenetic tree using the neighbor-joining method based on partial (175 nt) sequences of the virus small segment. Numbers in nodes indicate bootstrap values for the groups; values <75 are not shown. Strains detected from Spain are named by geographic origin, locality sampling site, and sampling year; other sequences are named by GenBank accession number, strain, geographic origin, and sampling year. Asterisks indicate sequences from this study that have been submitted to the EMBL (https://www.embl.org) and GenBank databases. Genotypes are indicated by Roman numerals: I, West Africa (Africa 1); II, Central Africa (Africa 2); III, South and West Africa (Africa 3); IV, Middle East/Asia, divided into groups corresponding to groups Asia 1 and Asia 2; V, Europe/Turkey (Europe 1); VI, Greece (Europe 2). Using guidelines published elsewhere (*25*,*26*), we then named and labeled the genotypes with equivalent clade nomenclature indicated in parentheses. Scale bar indicates substitutions/site (evolutionary distance).

We found CCHFV-positive feeding ticks in 128 (3.2%) of 3,959 pools (Table 3). We can therefore confirm that feeding ticks carried CCHFV RNA in 7 of 11 counties in 4 provinces in 4 regions: Cáceres (EXT), Madrid (MAD), Toledo (CLM), and Ávila (CyL) (Figure 1). We found marked differences in percentages of positive pools among regions: 1.5% in EXT, 0.3% in CyL, 11.8% in CLM, and 7.7% in MAD (Table 3). Ticks in which we detected CCHFV were mainly *H. lusitanicum*, although the virus was also found in *I. ricinus* (2 pools obtained from red deer), *R. annulatus* (1 pool from a fallow deer), and *D. marginatus* (1 pool from a wild boar) ticks. This finding does not indicate the vectorial characteristics of those tick species, because we collected them while they were feeding, but does indicate the breadth of distribution of the virus. With respect to the genetic variability of S segment sequences, in EXT we found genotypes I (Africa 1 clade), III (Africa 3 clade), and IV (Africa 4 clade) (Table 3; Figure 2); in MAD and CyL, we found genotype IV (Africa 4 clade), and in CLM, we found genotype III (Africa 3 clade).

### Questing Ticks

We studied 597 pools (1,791 ticks) collected while questing: 452 pools (1,356 ticks) collected in 2017 and 145 pools (435 ticks) in 2018 (Table 4). The 2 ticks not identified as *H. lusitanicum* were *H. marginatum.* We found CCHFV-positive *H. lusitanicum* ticks in 7 pools, all collected in 2 provinces of AND region, Córdoba and Cádiz (4.2% positive pools in both provinces), of genotypes III (Africa 3 clade) and IV (Africa 4 clade), determined according to their S segment sequences (Table 4; Figure 1, Figure 2).

## Discussion

After the diagnosis of the first human cases of CCHF in Spain in 2016 (*11*), a large field study was planned and implemented, with the support of human and animal health authorities, that aimed to estimate the geographic distribution of CCHFV in Spain. The virus is considered a serious human health issue, so risk assessment had to be based on a geographically wide-ranging campaign of viral detection, examining ticks collected while questing or feeding on diverse vertebrates. By testing for CCHFV RNA, we used ticks as sentinels for the presence of the virus. Although the study was not designed to estimate prevalence, our data showed a 2.96% rate of positivity (135 positive pools out of 4,556), close to the values reported from other endemic locations, such as Turkey (3.6%), Albania (3.2%), and Kosovo (3.6%) (*27*–*29*). However, the wide range of methods used to collect ticks and analyze samples make reliable comparisons difficult.

In total, 3,959 pools were processed from 10,793 collected ticks. Tick management with acaricide is routinely practiced with domestic animals so they rarely have ticks; therefore, most of the feeding ticks were collected from wild animals. All of the positive tick pools were obtained from wild ungulates, consistent with the higher CCHFV antibody prevalence found in wildlife (61%) compared with livestock (15%) in the same areas (*30*). Wild ungulates range freely in most of the studied territory, although some are farmed as game, where the animals are confined to large farms. On these farms, game animals can be exposed to immature *Hyalomma* ticks through close contact with prominent hosts, such as rabbits and hares, simultaneously explaining the greater abundance of infected ticks and the higher serologic titers among farmed wild ungulates than among livestock.

The significance of finding CCHFV in feeding ticks is always difficult to interpret, because the virus could have been acquired through the blood meal, which means that determining the ticks’ CCHFV status cannot provide an accurate estimate of actual infection rates. We used the feeding ticks as sentinels of the presence of CCHFV and as a means of comparing the prevalence of the virus noted in questing ticks. Although collecting questing ticks is always more expensive and time-consuming, combining data from both sources could help provide a more balanced perspective for large geographic areas such as entire countries.

We found CCHFV from *I. ricinus*, *Rhipicephalus* spp., and *D. marginatus* ticks in 4 pools, but we considered these data to reflect serendipitous detection of viral RNA in feeding ticks. All of our results point toward a predominant role for *H. lusitanicum* in CCHFV circulation. In fact, it is very likely that circulation of the virus is restricted to *H. lusitanicum* ticks, a factor that had been suspected (*20*,*21*) but not confirmed. This finding emerged from the second phase of the study, in which we surveyed questing ticks. We demonstrated that the virus can successfully complete cycles in nature while perpetuating itself in *H. lusitanicum* ticks. This finding adds an extra dimension to our results, because the range of *H. lusitanicum* ticks is rapidly expanding as a consequence of the spread of one of its natural hosts, wild boars. Although the vectorial status of this tick has not been demonstrated, the discovery of viral RNA in molted ticks after a blood meal demonstrates that the virus can at least persist in these ticks (*5*). It is therefore a matter of urgency to establish the vectorial status of the species, as well as its preferences for biting humans, which seems to be a promising field of research. Because this tick is a potential source of CCHFV infection, its ability to be a parasite in humans has clearly been neglected, probably because it has not been reliably identified in the few samples collected from humans. 

We identified 5 regions in central and southwest Spain where CCHFV is present. Taken together, findings from our report and an earlier study (*21*) indicate the permanent circulation of CCHFV in these regions, which are characterized by a Mediterranean forest ecosystem rich in wild ungulates that undoubtedly favors the presence of *H. lusitanicum* ticks. The lack of positive detection in MUR, one of the surveyed regions, does not prove that the virus is not present there, but more likely reflects the small number of ticks collected from the region. 

Our system of confirmation involved amplification by 2 methods or from 2 extractions amplified using the same method. We confirmed positive amplification by real-time or nested RT-PCR in 118 out of 135 pools. We could not confirm amplification in the other 17 pools and had to repeat extraction and real-time RT-PCR. Given that sensitivity in the 2 PCR methods is similar, these differences in amplification could be because of variability in the target region (*31*). We obtained sequences from 105 of 128 pools of ticks collected from animals and for 3 of 7 pools collected from vegetation, which enabled us to identify genotypes on the basis of their S segment sequences. Viruses belonging to Africa 3 (genotype III) and Europe 1 (genotype V) had previously been detected in ticks; the newly proposed Africa 4 clade should be added to these findings (*18*–*21*) and should be interpreted as arising through the continuous exchange of infected ticks by birds migrating between Africa and southern Europe. This survey also detected the circulation of Africa 1 (genotype I) in Spain. These results confirm the wider-than-expected distribution and broad variability of CCHFV in Spain. These findings were initially unexpected but are compatible with reports of the genetic variability of the virus, because CCHFV is well known to undergo rearrangement to produce diverse combinations of the S, L, and M segments (*32*).

The relationship of genotypes III, IV, and V to human cases in Spain has previously been described (Africa 3 in cases from 2016 and Africa 4 and Europe 1 from cases in 2018) (*13*,*14*,*17*). The great variability of genotypes may have resulted from multiple introduction events in Spain, but the complete mechanism of spread is very poorly understood. Published syntheses have reported that immature *H. lusitanicum* ticks feed on small mammals, perhaps mainly leporids, and adults feed on large ungulates (*6*). However, previous studies (*33*–*36*) have documented that when there are huge populations of the tick, large numbers of immature ticks were found on birds, such as the red partridge (*Alectoris rufa*), that spend most of their time on the ground.

Parasitism of birds does not seem to be the rule for *H. lusitanicum* ticks, which have not been found on birds migrating from Africa to Europe. We consider birds to be secondary, or even accidental, hosts for immature *H. lusitanicum* ticks, and therefore that immature *H. marginatum* ticks, which commonly feed on birds, may be the keystone species for transporting and importing CCHFV. We propose that annual migratory journeys from Africa of birds carrying *H. marginatum* ticks may have been the primary source of entry for several viral variants into Spain. Once introduced, the virus could have easily adapted to a cycle of transmission between wild ungulates and *H. lusitanicum* ticks, probably acquiring new mutations or reassortments. This hypothesis will be difficult to prove unless more data about *H. marginatum* ticks transported from Africa become available. Furthermore, the presence of European viral genotypes is difficult to reconcile with our observations and contrasts with data from other countries, such as Turkey and those of the Balkan region, where the virus is endemic. The occurrence of only 1 introduction from Asia to these countries has been proposed, and the strains causing human cases there have remained genetically stable for decades (*37*).

Our findings on the distribution of CCHFV in Spain demonstrate its presence in 5 regions covering the central and southwest part of the country. Our study also drew attention to the importance of *H. lusitanicum* ticks in circulating the virus including several viral genotypes and possible new reassortments. The risk for transmission to humans has not yet been possible to calculate because of the paucity of data. Research is needed to determine the reasons behind the high variability of CCHFV and the actual distribution and origin of circulating strains.

Clinicians, especially general practitioners, as well as laboratory staff, public health workers, stakeholders, and the general public need to be aware of the situation regarding CCHFV in Spain. Because some clinical cases may be mild and etiologically unresolved by practitioners, suitable tools must be made available that can detect the virus in suspected clinical cases in Spain. Diagnosis of CCHF is hampered by the biosafety conditions required to manage a virus of high biologic risk.

Public health activities, including surveillance of zoonoses like CCHF, need to be carried out under the One Health umbrella, as was done in our study. Large-scale seroprevalence studies in animals and humans are currently underway. The huge effort required to coordinate local and national public health representatives and entomologists, virologists, and animal and human health specialists should be an essential step in the control of these pathogens. 
